# Multimodality imaging assessment of primary pericardial rhabdomyosarcoma: a case report

**DOI:** 10.3389/fcvm.2023.1237951

**Published:** 2023-08-14

**Authors:** Xingxuan Li, Yukun Cao, Guozhu Shao, Yue Cui, Yumin Li, Kailu Zhang, Xiaoqing Liu, Heshui Shi

**Affiliations:** ^1^ Cancer Center, Union Hospital, Tongji Medical College, Huazhong University of Science and Technology, Wuhan, China; ^2^ Department of Radiology, Union Hospital, Tongji Medical College, Huazhong University of Science and Technology, Wuhan, China

**Keywords:** cardiac tumor, rhabdomyosarcoma, cardiac magnetic resonance, PET-CT, echocardiography

## Abstract

Primary pericardial sarcomas are rare and lethal diseases. To date, only a few cases of primary pericardial sarcomas, such as rhabdomyosarcoma (RMS), have been reported. Since the unusual location of RMS in the pericardium makes it challenging to diagnose, precise diagnostic procedures are required. In this study, we present the case of a 23-year-old man who experienced postprandial obstruction and atypical precordial pain that lasted for a week. Echocardiography revealed a heterogeneous isoechoic pericardial mass with a significant pericardial effusion. Contrast-enhanced CT revealed a massive pericardial effusion along with an irregular, defined, heterogeneously enhancing mass that was located between the pericardium and diaphragm. PET-CT imaging showed an intense FDG uptake in the pericardial mass. Furthermore, cardiac MRI demonstrated malignant characteristics of the pericardial mass and provided a detailed visualization of its exact anatomical connection with both cardiac and extracardiac structures. Finally, a pathologic examination of a puncture biopsy specimen confirmed the diagnosis of primary pericardial RMS. Our case emphasizes the importance of multimodal imaging for the differential diagnosis and evaluation of cardiac involvement, while providing clinicians with crucial information for clinical treatment and decision-making.

## Background

Despite being rare, cardiac masses remain a significant part of cardio-oncology in clinical practice. These masses include benign tumors, primary or secondary malignant tumors, and tumor-like masses. The prevalence of primary cardiac tumors is reported to range from 0.001% to 0.3% (1). However, primary pericardial tumors are much rarer than primary cardiac tumors, accounting for only 0.001% to 0.007% (2). These tumors are predominantly malignant, with more than half of cases seen in younger patients, typically between the ages of 20 and 30, and are associated with a poor prognosis (3). The gold standard for treating malignant pericardial tumors is complete surgical resection. However, because of the aggressive nature of the tumors, more than 40% of patients may present with metastatic conditions (1).

With the advent of multimodal imaging, it is now possible to comprehensively identify the etiology of cardiac masses in many cases, in conjunction with clinical information (4). In this article, we report a case to highlight the importance of multimodal imaging in precise diagnosis and management of a rare primary pericardial sarcoma.

## Case presentation

A 23-year-old male patient was referred to our hospital clinic due to symptoms of postprandial obstruction and atypical precordial pain that persisted for one week (Supplementary Table S1). He had no significant medical history relevant and no past interventions. On presentation, physical examination revealed a heart rate of 61 beats per minute, a blood pressure of 122/62 mmHg, and normal oxygen saturation. Chest auscultation revealed clear lungs and no cardiac murmur, and there was no lower extremity edema or jugular vein dilatation.

The resting electrocardiogram indicated sinus rhythm and ST-segment elevation (Supplementary Figure S1). Initial blood tests revealed mild elevation in B-type natriuretic peptide (BNP) at 188 pg/ml (normal range <100 pg/ml), aspartate aminotransferase at 67 U/L (normal range 8–40 U/L), and lactate dehydrogenase at 306 U/L (normal range 109–245 U/L). The levels of D-dimer, fibrinogen degradation products, and a tumor marker known as neuron-specific enolase were highly elevated at 7.95 mg/L (normal range <0.5 mg/L), 28.8 ug/ml (normal range <5 ug/ml) and 24.67 ug/L (normal range <16.3 ug/L), respectively.

**Figure 1:**
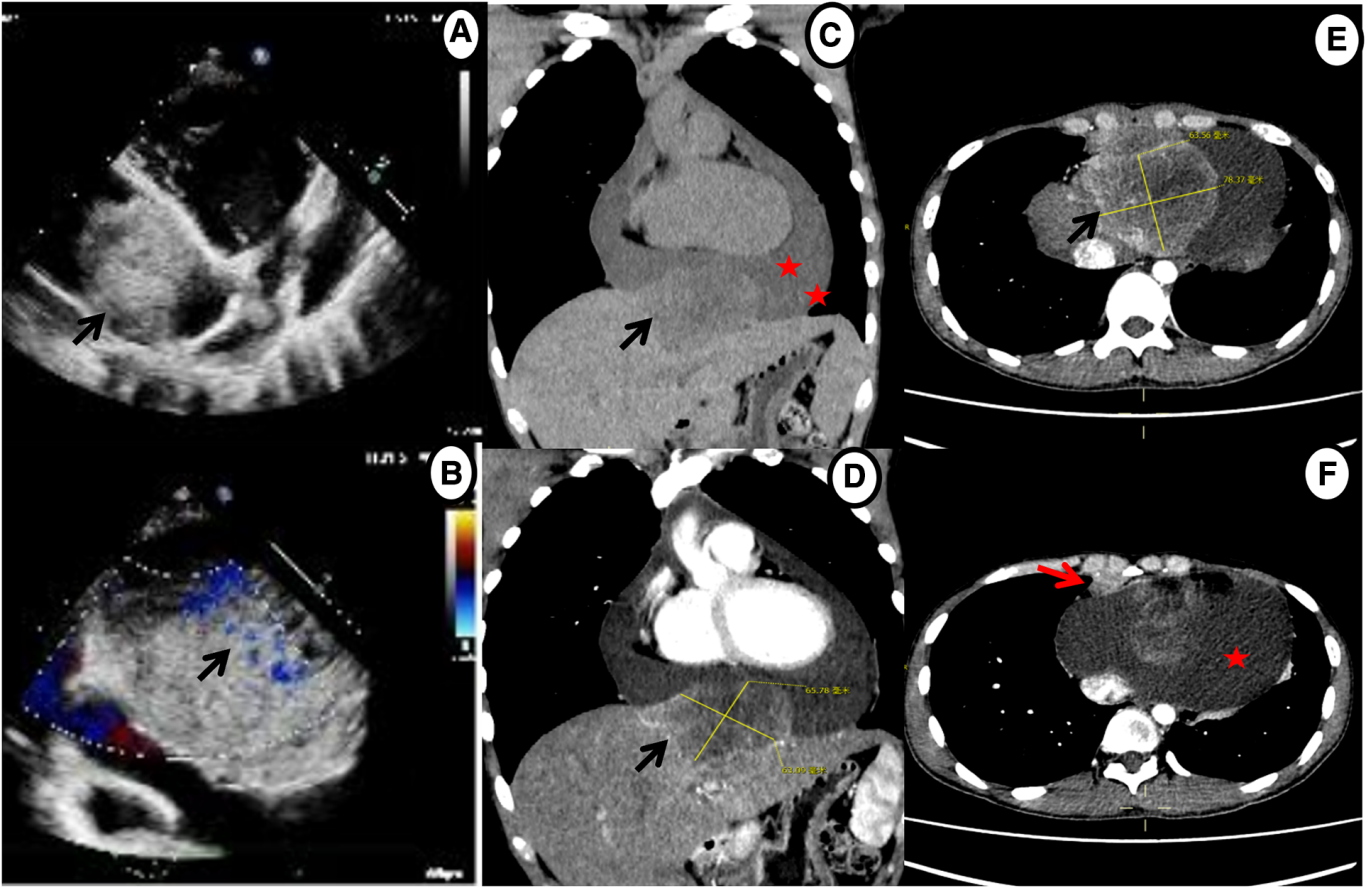
Echocardiography and chest contrast-enhanced CT demonstrating a massive mass (black arrow) and pericardial effusion (star). (**A**,**B**) Echocardiography shows a mass in the pericardium with a small amount of blood flow signals. (**C–E**) CT reveals an irregular, heterogeneously enhancing mass between pericardium and diaphragm with massive pericardial effusion. (**F**) Axial contrast-enhanced CT shows enlarged enhancing lymph nodes in the right cardio-diaphragmatic angle (red arrow).

Given his unusual symptoms and laboratory results, transthoracic echocardiography (TTE) and chest contrast-enhanced computed tomography (CT) were recommended. The TTE revealed a heterogeneous isoechoic pericardial mass with massive pericardial effusion (Figure 1A). Color Doppler flow imaging demonstrated minimal blood flow signals along the margins of the mass (Figure 1B). The dimensions of the atria and ventricles appeared to be within the normal range, and the morphology and function of the cardiac valves were unremarkable. The CT scan revealed a 6.5 cm × 7.8 cm × 6.3 cm irregular mass between the pericardium and diaphragm and significant pericardial effusion (up to 40 mm) (Figures 1C–E). Additionally, a 1.0 cm enlarged lymph node was detected in the right cardio-diaphragmatic angle (Figure 1F).

**Figure 2:**
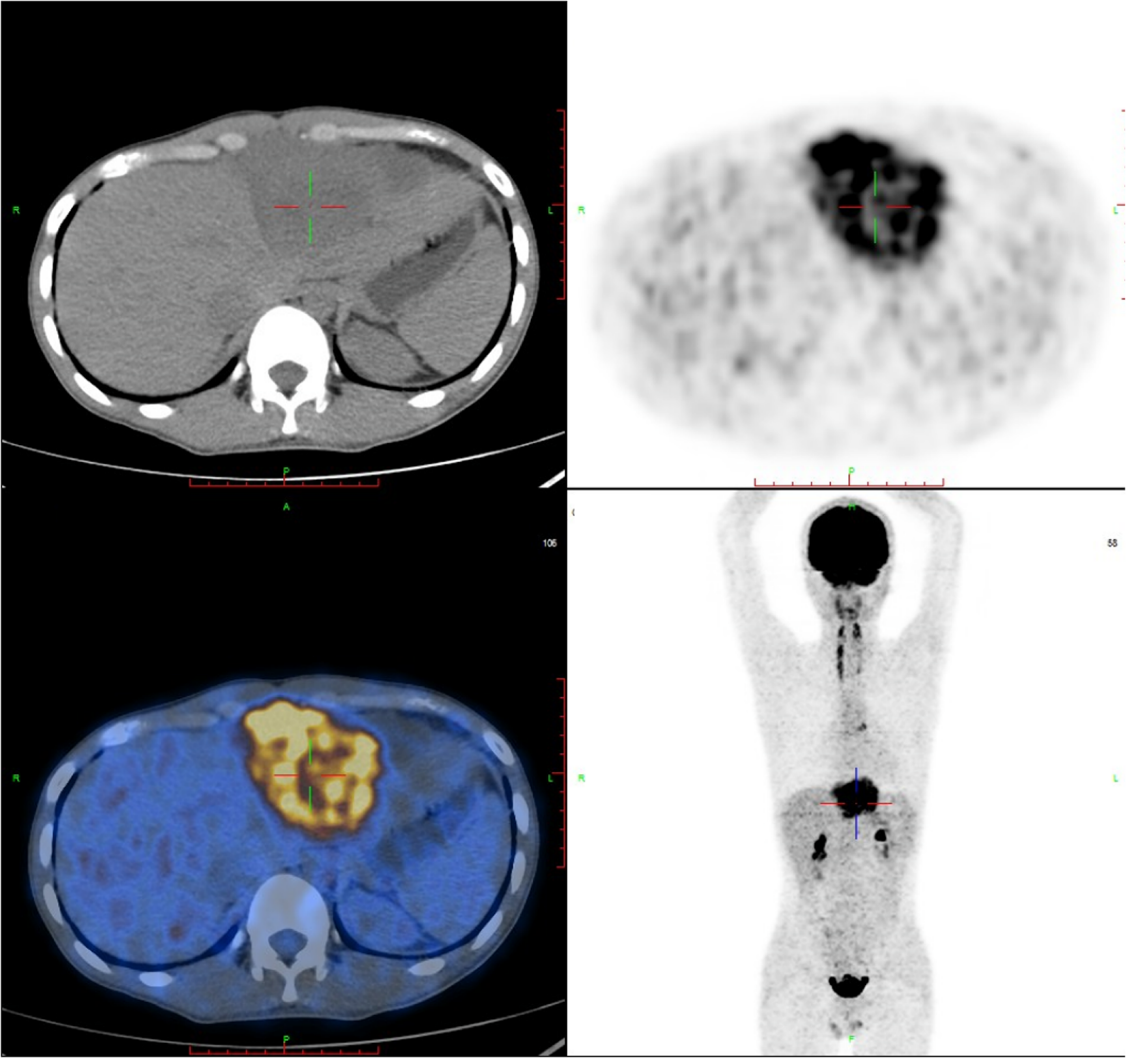
FDG-PET reveals hypermetabolic activity located between the pericardium and diaphragm, with a standard uptake value (SUVmax) ranging from 5.4–15.9, and no abnormal lesions are found in other parts of the body. FDG-PET, Fluorine-18 fluorodeoxyglucose positron emission tomography.

A F18-fluorodeoxy glucose-positron emission tomography (18F-FDG PET-CT) scan was recommended to provide additional information about the mass and evaluate its metabolic activity. The PET-CT scan revealed hypermetabolic activity located between the pericardium and diaphragm, with a standard uptake value (SUVmax) ranging from 5.4 to 15.9 (Figure 2). Additionally, hypermetabolic activity was detected in the lymph nodes of the right cardio-diaphragmatic angle, with SUVmax values of 5.9–7.3. No hypermetabolic lesions were detected in other parts of the body.

**Figure 3:**
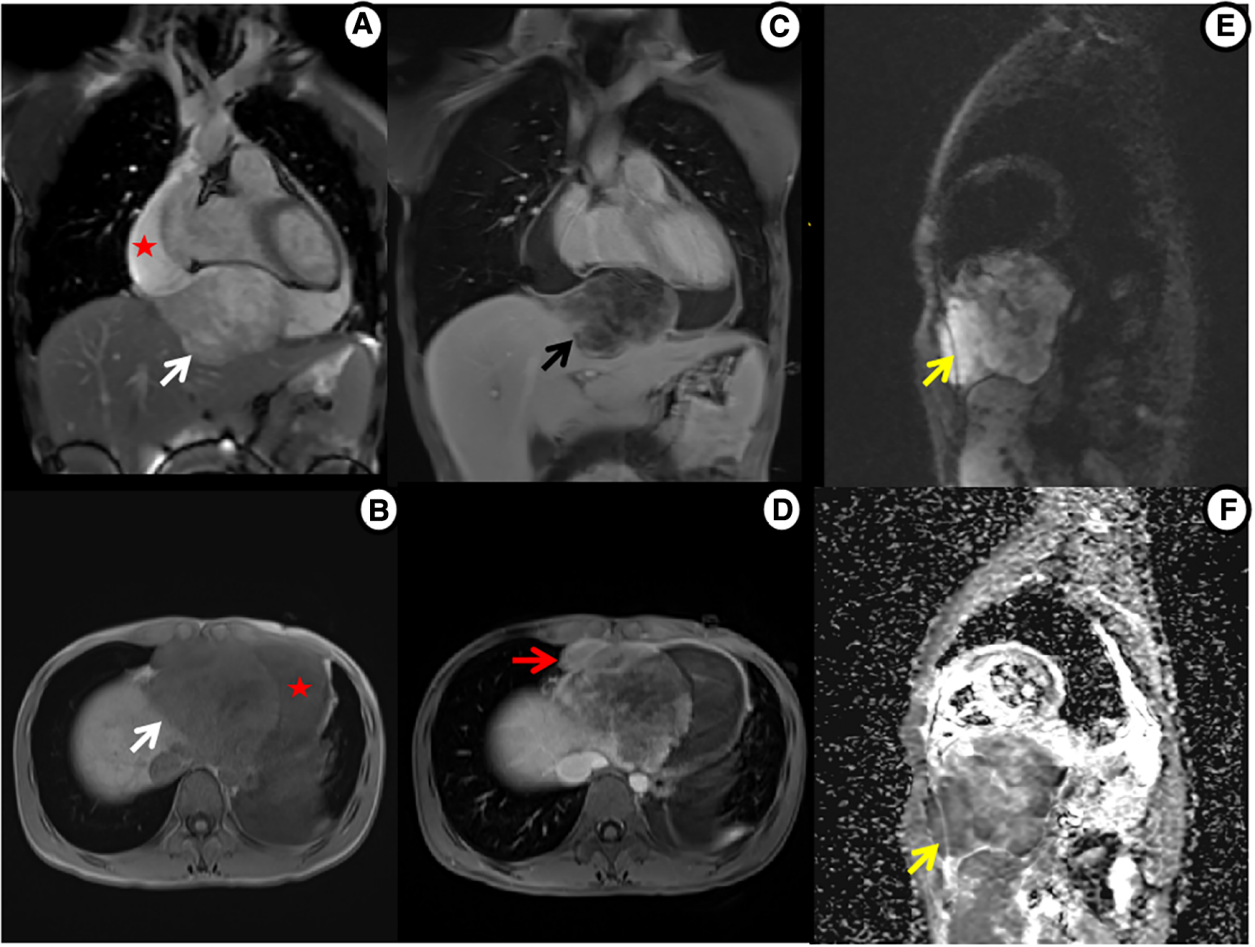
Cardiac MRI shows mass tissue characterization. (**A**) Cine imaging: heterogeneously hyperintense mass (white arrow) and large pericardial effusion (star). (**B**) Precontrast T1WI: slightly hypointense mass. (**C**) Postcontrast T1WI: non-enhanced core within mass, surrounded by slightly heterogeneous enhancement (black arrow). (**D**) Postcontrast T1WI: enlarged enhanced lymph nodes (red arrow). (**E,F**) Diffusion MRI: bright mass on DWI and dark on ADC (yellow arrow).

**Figure 4:**
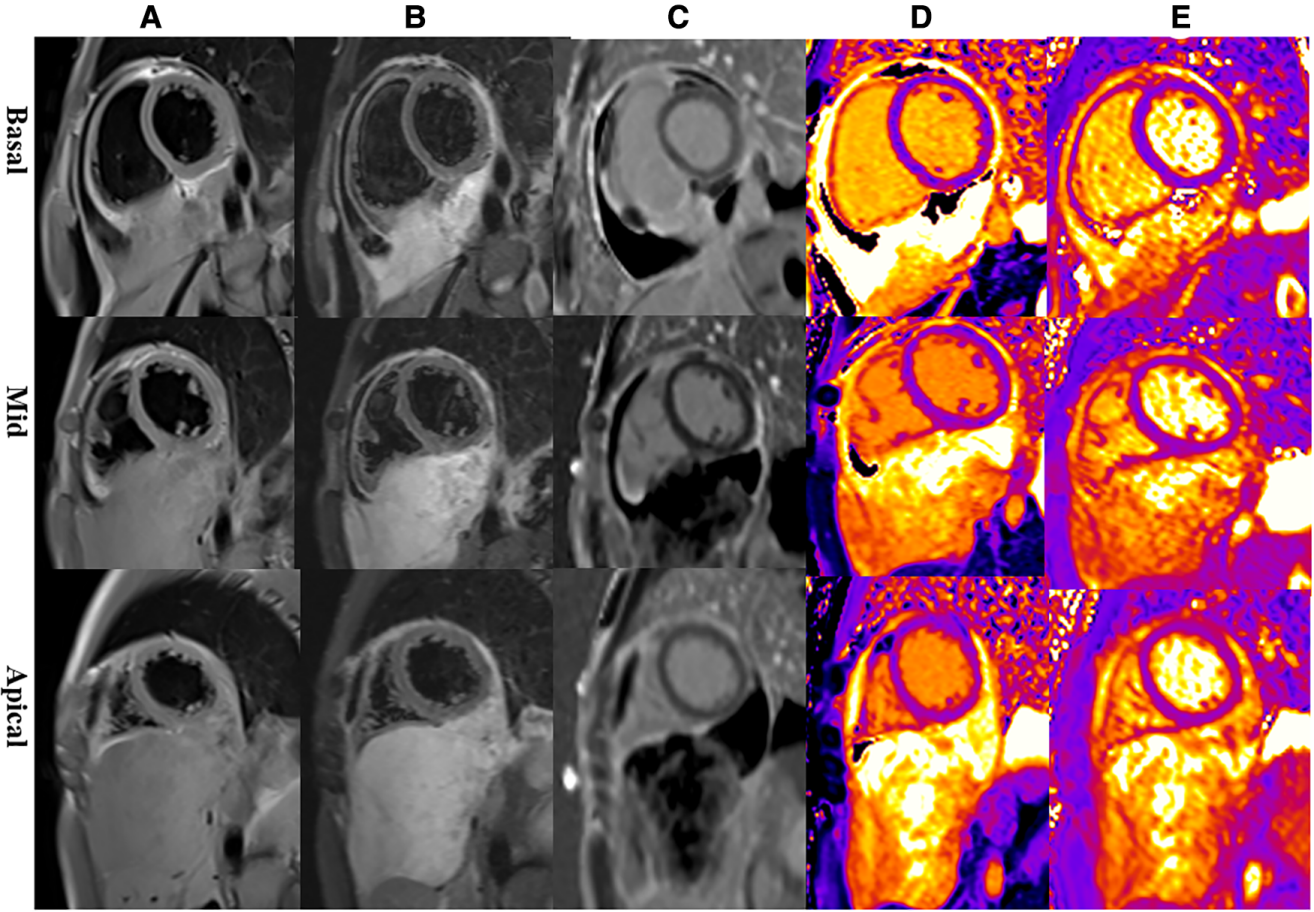
Representative magnetic resonance images of the basal, mid, and apical short-axis slices. (**A**) Dark-blood T1-weighted images show isointense mass. (**B**) Dark-blood T2-weighted images show strongly hyperintense mass. (**C**) Late gadolinium enhancement sequences reveal that there is peripheral heterogeneous uptake and a spared central core. (**D**) Native T1 mapping depicts evidently elevated T1 values in the mass. (**E**) T2 mapping depicts evidently elevated T2 values in the mass.

Multiparametric cardiac magnetic resonance imaging (CMR) further showed signal features suggestive for a malignant nature of the pericardial mass. Specifically, in accordance with CT, CMR showed an irregular defined mass between the pericardium and diaphragm, adjoining with enlarged lymph nodes in the right cardio-diaphragmatic angle and massive pericardial effusion (Figure 3). In cine imaging using a steady-state free-precession sequence, slight motion amplitude was observed in the large pericardial mass. However, there was no effect on the contractility of the global myocardium (Supplementary Video S1–S2). The relaxation of the inferior wall of the biventricle was slightly limited due to the compression of the adjacent myocardium by the mass (Supplementary Video S3). The analysis of cardiac function revealed preserved biventricular systolic function, with left ventricular ejection fraction at 69% and right ventricular ejection fraction at 63%. Despite the normal LV ejection fraction but elevated BNP, tissue tracking technology was used to conduct myocardial strain analysis (Supplementary Figure S2). The results indicated a decrease in the global longitudinal strain of the left ventricle (−11%; reference range: −15%–18%), while the global radial (34%; reference range: 30%–37%) and circumferential (−24%; reference range: −21%–25%) strains remained normal. These findings suggest the possibility of subtle segment contractile dysfunction due to the presence of a pericardial inflammation or a mass between the myocardium and pericardium. A conventional plain scanning sequence demonstrated a slightly hypointense appearance on T1w-imaging and a heterogeneously hyperintense appearance on T2w-imaging of the mass (Figures 3A,B, 4A,B). The presence of heterogeneously strongly elevated T1 (1,700–2,500 ms) and T2 values (120–172 ms) within the mass was also documented using T1 and T2 mapping images (Figures 4D,E). However, the native T1 (908 ms), T2 (46 ms) and extracellular volume (25%) values of left ventricular myocardium were approximately normal. Resting first-pass perfusion showed that there was a complete perfusion defect within the mass related to avascularity (Supplementary Video S4). On late gadolinium enhancement (LGE) imaging, there was no LGE core within the mass, surrounded by slightly heterogeneously high signal enhancement (Figures 3C,D, 4C). Diffusion MRI revealed that the mass was homogeneously bright on diffusion weighted imaging (DWI) and dark on apparent diffusion coefficient (ADC) (Figures 3E,F), suggestive of reduced diffusivity possibly owing to hypercellularity.

In addition, a CT-guided percutaneous needle biopsy was conducted, and subsequently, histopathological analysis of the tissue sample supported the diagnosis of pericardial sclerosing rhabdomyosarcoma (RMS) (Supplementary Figure S3). The immunohistochemical results were as follows: CD99 (+), Desmin (+), MyoD1 (diffusely strong+), Myogenin (focal+), Vimentin(+), WT1 (Paranuclear+), D2-40 (+), CD56 (+), FLi-1 (+), ALK (+), MDM2 (+), CDK4 (+), P16 (focal+), S100 (partly mild+), Ki67 (LI: approximately 30%). Due to the tumor's size, location, and malignancy grade, the patient was referred to the oncology department of our hospital for conservative chemotherapy. However, during the eight-month follow-up after treatment, both the mass and lymph nodes in the right cardio-diaphragmatic angle showed modest enlargement (Supplementary Figure S4).

## Discussion

Primary pericardial sarcomas are a rare and fatal type of cancer, accounting for about 10% of all primary cardiac sarcomas (5). Unlike other sarcomas, RMS tumors in the pericardium typically arise from the myocardium and have a propensity to involve multiple areas within the heart. However, primary pericardial involvement in patients is extremely rare (1). These cases are most frequently observed in infants and children, and there are no documented instances of RMS in young adult patients thus far (2, 3).

Due to its association with pericardial effusion, pericarditis, or invasion of adjacent structures, the clinical symptoms and signs of pericardial RMS are usually nonspecific. As a result, patients often present with a variety of symptoms, such as chest pain, dyspnea, and palpitations, often accompanied by pericardial effusion (6). In the current case, the patient reported feeling of obstruction after eating and atypical precordial pain, which was likely associated with the tumor's involvement of the adjacent diaphragm and pericardium. The ECG showed ST-segment elevation, which could be attributed to pericarditis caused by the mass invading the inferior pericardium, myocardial ischemia, or nonspecific physiological factors. Because of its rapid growth rate prior to clinical manifestation, RMS is challenging to treat and carries a grim prognosis. The gold standard of sarcoma treatment is surgery; however, the effectiveness of surgical removal is dependent on the tumor's anatomical location and metastasis. In primary pericardial RMS, the standard therapy in recent decades has been a combination of surgery, chemotherapy, and radiation therapy. Nonetheless, immediate surgical intervention was not recommended for the patient due to the following reasons: extensive tumor invasion of the diaphragm and potential lymph node metastasis in the right cardio-diaphragmatic angle. As a result, the patient underwent primary systemic chemotherapy after admission.

TTE has the capability to assess the impact of mass on both cardiac function and morphology. Additionally, it is also able to identify space-occupying lesions. However, TTE has several limitations, including a lower signal-to-noise ratio, weak acoustic windows, and through-plane motion artifacts. Cardiac CT offers valuable insights into the morphology of tumors with exceptional spatial and temporal resolution. PET provides a precise evaluation of the metabolic activity of tumors by using 18F-FDG. FDG-PET assists in staging malignancies and also aids in the identification of possible myocardial and pericardial involvement. It is a valuable tool for assessing early responses to cancer treatment, planning radiation therapy, and determining optimal biopsy location (7). Nevertheless, a limitation of FDG-PET is the requirement for dietary preparation, particularly in patients with intra-myocardial tumors and those with a history of radiation exposure (8).

Cardiac MRI is a highly valuable diagnostic imaging technique for assessing and follow-up in patients with cardiac masses (9). It offers multiplanar imaging with a wide field of view, without exposing patients to ionizing radiation. Additionally, it allows for the assessment of cardiac functional and anatomical characteristics, as well as myocardial tissue characterization (10). This information is critical for the early diagnosis and timely treatment of patients. Supplementary Table S2 presents a summary of the clinical and imaging features reported in primary pericardial sarcomas from published case reports (9–21). Our analysis reveals that these tumors tend to be large, with irregular or ill-defined borders, particularly when their size exceeds 5 cm (14, 15, 17, 18). Furthermore, central areas of necrosis or hemorrhage may be evident, and there is often a significant degree of pericardial effusion or hemopericardium. Its enhancement features are closely associated with vascularity and pathological components (10, 12, 21). The patient showed ECG abnormalities and BNP elevation. Myocardial strain analysis revealed a decrease in the global longitudinal strain of the left ventricle, while the native T1, T2, and extracellular volume values of the left ventricular myocardium were within normal range. Hence, we postulate that the elevation of BNP is associated with abnormal left ventricular myocardial strain.

In our case, the enhanced CT and PET imaging revealed atypical features of heterogeneous attenuation, enhancement, and hypermetabolic activity. However, these findings complicate the process of making a differential diagnosis of the tumor. After a thorough review of the pericardial RMS patient's multiparametric CMR imaging, the following features have been identified: (1) heterogeneous hypointensity on T1-weighted images and hyperintensity on T2-weighted images indicate tumor tissue characteristics and part necrosis; (2) T1 and T2 mapping images with elevated T1 and T2 values reveal specific fibrosis and edema within the mass; (3) resting first-pass perfusion imaging shows a complete hypointense defect, indicating avascularity within the mass; (4) slightly heterogeneous LGE is associated with fibrosis and partial necrosis; (5) bright DWI and dark ADC in diffusion MRI suggest malignant tendencies; (6) cine and conventional plain scan sequences contribute to the description of its association with cardiac and extracardiac structures. Nonetheless, regarding the CMR features of pericardial RMS, only two cases have been reported, and the CMR images are deficient in providing elaborate details. In addition, it is a rare malignant tumor mostly found in children and not adults; therefore, limited data hinders our understanding of the multiparametric features of pericardial RMS in adults.

## Conclusion

Pericardial RMS is an exceptionally uncommon disease with an unfavorable result. Early diagnosis and timely intervention could potentially improve the prognosis. We described a case of atypical presentation of pericardial RMS and its multimodal imaging features. Multimodality imaging techniques, particularly cardiac MRI, allow for the evaluation of the size, borders, and tissue characteristics of the cardiac mass. It also helps in distinguishing its relationship with cardiac and extracardiac structures, quantifying its vascularity and degree of enhancement, and assessing its impact on cardiac structure and function. Nonetheless, further experience is necessary to gain a better understanding of the imaging characteristics of primary malignant pericardial tumors.

## Data Availability

The original contributions presented in the study are included in the article/**Supplementary Material**, further inquiries can be directed to the corresponding authors.
