# Supplementary material for: Multimodality imaging assessment of primary pericardial rhabdomyosarcoma: a case report
Source: Front Cardiovasc Med. 2023 Aug 14;10:1237951. doi: 10.3389/fcvm.2023.1237951 (PMC10461312; doi:10.3389/fcvm.2023.1237951)
Supplement: Supplementary file 1 [file Presentation1.zip › Supplementary material/Supplementary Figure S1-4.docx]

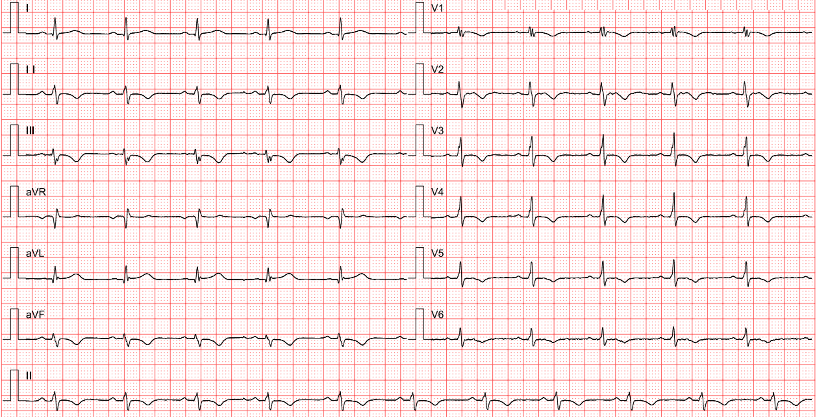


**Supplementary Figure S1** Twelve-lead ECG findings on admission


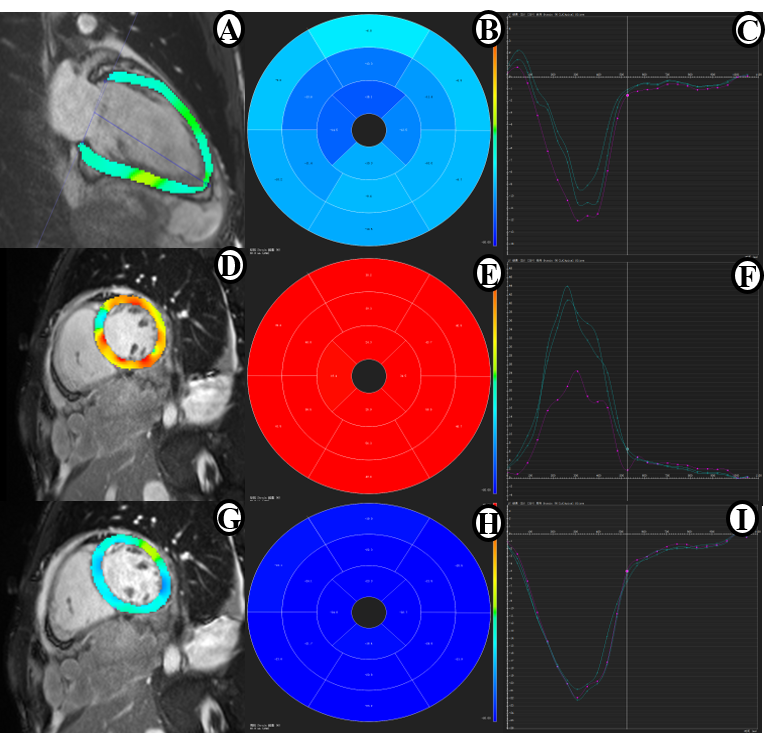


**Supplementary Figure S2** Diagram of the left ventricular myocardium strain analysis. Representative contour of the endocardium and epicardium of the left ventricle of longitudinal (A), radial (D), and circumferential (G) strain analysis are shown in the left column. The longitudinal (B), radial (E), and circumferential (H) strain values in a 16-segment model are presented in the middle column. The longitudinal (C), radial (F), and circumferential (I) strain–time curves are shown in the right column.


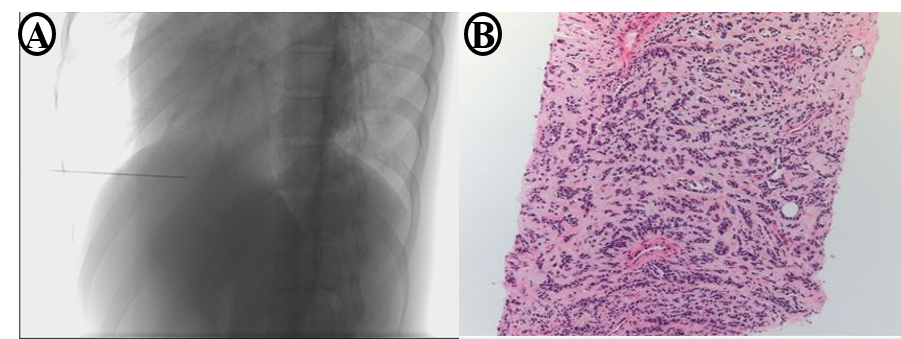


**Supplementary Figure S3**: Representative image of CT-guided percutaneous needle biopsy. B, Histopathological examination demonstrates sclerosing rhabdomyosarcoma of the tumor.


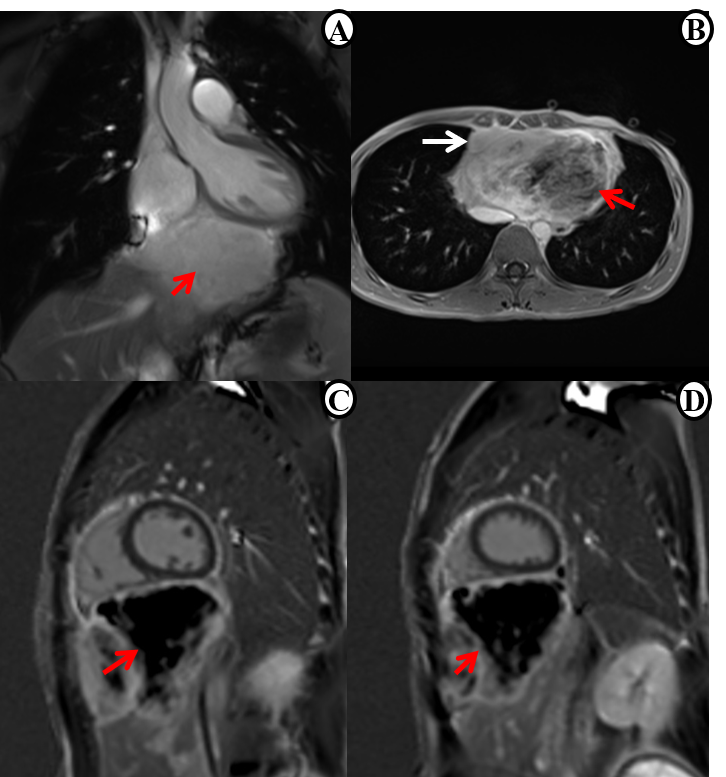


**Supplementary Figure S4**  At the 3-month follow-up after chemotherapy, a representative cine image (A), Postcontrast T1-weighted image (B), and late gadolinium enhancement of short-axis slices (C and D) show that both the mass (red arrow) and lymph node (white arrow) in the right cardio-diaphragmatic angle were slightly enlarged (baseline size of the mass: 6.5×7.8×6.3 cm, 8-month follow-up: 7.2×11.0×8.2 cm; baseline short diameter of the lymph node:1.0 cm, 8-month follow-up:1.6 cm ).
