# Supplementary material for: Multimodality imaging assessment of primary pericardial rhabdomyosarcoma: a case report
Source: Front Cardiovasc Med. 2023 Aug 14;10:1237951. doi: 10.3389/fcvm.2023.1237951 (PMC10461312; doi:10.3389/fcvm.2023.1237951)
Supplement: Supplementary file 1 [file Presentation1.zip › Supplementary material/SupplementaryTable S1-2.DOCX]

**Supplementary Table S1：Timeline of the patient’s clinical course.**

| **April 07, 2022** | The patient was referred to our hospital clinic due to symptoms of postprandial obstruction and atypical precordial pain that persisted for one week |
| --- | --- |
| **April 07, 2022** | ECG indicated sinus rhythm and ST-segment elevation |
| **April 08, 2022** | Initial blood tests revealed mild elevation in BNP and NSE |
| **April 12, 2022** | TTE revealed a heterogeneous isoechoic pericardial mass with massive pericardial effusion |
| **April 16, 2022** | CT revealed an irregular mass between the pericardium and diaphragm and significant pericardial effusion |
| **April 19, 2022** | PET-CT revealed hypermetabolic activity located between the pericardium and diaphragm |
| **April 21, 2022** | Cardiac MRI showed malignant characteristics of the mass and provided a detailed visualization of its exact anatomical connection with both cardiac and extracardiac structures. |
| **April 28, 2022** | The tissue sample histopathological analysis supported the diagnosis of pericardial RMS |
| **May 10, 2022** | Due to the tumor's size, location, and malignancy grade, conservative chemotherapy was performed |

ECG, electrocardiogram; BNP, B-type natriuretic peptide; NSE, neuron-specific enolase; TTE, transthoracic echocardiography; CT, computed tomography; PET, positron emission tomography; MRI, magnetic resonance imaging; RMS, rhabdomyosarcoma

**Supplementary Table S2：Imaging features in patients with primary malignant pericardial tumors**

| Tumor | Common Age | Common Sex | Morphologic features | Pericardial effusion | CT features | CMR features | | |
| --- | --- | --- | --- | --- | --- | --- | --- | --- |
|  |  |  |  |  |  | T1WI* | T2WI* | LGE |
| Mesothelioma | 55 years | M:F=1.5-2:1 | multiple and ill-defined, diffuse growth | moderate to massive  or diffuse thickening | diffuse pericardial thickening, nodular enhancement,atypical calcification | Isointense | Heterogeneous | Heterogeneous |
| Angiosarcoma | 30-50 years | M: F =3:1 | Large and ill-defined, broad base, hemorrhage and necrosis | Massive or diffuse thickening | Heterogeneous  attenuation | heterogeneous and variable | Heterogeneous and hyperintense | avid enhancement with “sun ray” |
| Synovial Sarcoma | 33 years | M: F=4.28:1 | Smooth and solid masses, infiltrating myocardium or encasing vessels | Massive | Low attenuation | Isointense | Slightly hyperintense | Heterogeneous |
| Fibrosarcoma | 16-32 years | - | Infiltrative, central areas of necrosis | Massive | Heterogeneous  attenuation | heterogeneous | heterogeneous | Heterogeneous |
| Liposarcoma | 22-69 years | M:F=1:2 | large size and invasive nature | Massive | predominantly fatty to heterogeneous | High  heterogeneous | High  heterogeneous | No/minimal uptake |
| Rhabdomyosarcoma | Infants, children | - | large, solid, infiltrative;  central necrosis and cavitation | Massive | Low attenuation | Isointense | Hyperintense | Heterogeneous enhancement |
| Lymphoma | 63 years | M: F =3:1 | Ill defined, infiltrative mass or  nodule | Massive, commonly hemorrhagic | iso-to hypoattenuating | iso- to hyperintense | Slightly hyperintense | Heterogeneous enhancement |

CMR, cardiac magnetic resonance; M, male; F, female; T1WI, T1-weighted imaging; T2WI, T2-weighted imaging; LGE, late gadolinium enhancement .

*T1WI and T2WI signal intensity is given relative to myocardium.
